# Supplementary material for: Identification of photoperiod-induced specific miRNAs in the adrenal glands of Sunite sheep (Ovis aries)
Source: Front Vet Sci. 2022 Jul 22;9:888207. doi: 10.3389/fvets.2022.888207 (PMC9354845; doi:10.3389/fvets.2022.888207)
Supplement: Supplementary file 1 [file Data_Sheet_1.ZIP › Supplementary file/miRDeep2 file.zip!/miRDeep2 file/Know_Structure/SP42A1/oar-mir-411a.pdf]

miRBase precursor	: oar-mir-411a
Total read count	: 32134
oar-miR-411a-5p read count	: 29648
oar-miR-411a-3p read count	: 2484
remaining reads	: 2

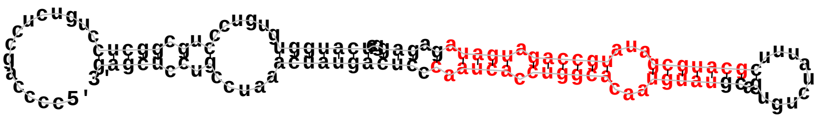

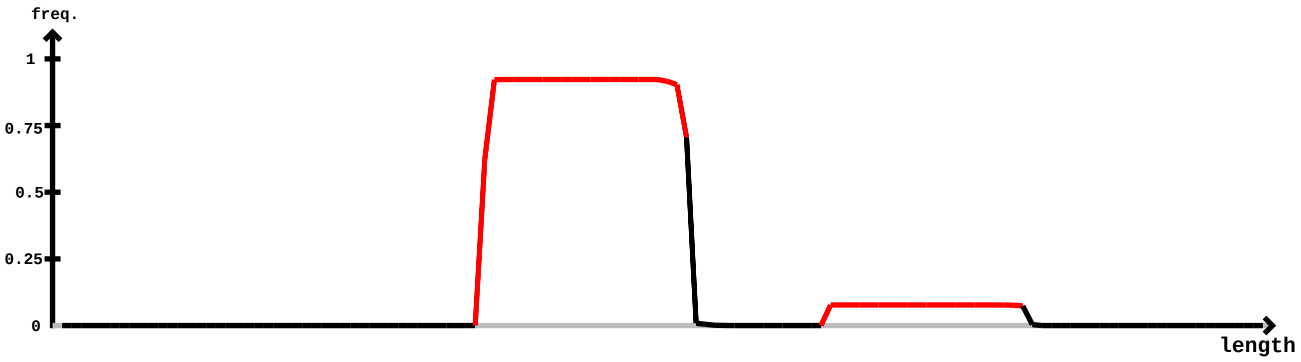


```
                                                                                    oar-miR-411a-3p
                                          oar-miR-411a-5p
5'- ccccagccucuguccucggcgucccugugugguacucggagagauaguagaccguauagcguacgcuuuaucugugacguauguaacacgguccacuaacccucaguaucaaauccguccucgag  -3'   exp
    ..............(((((.(.(......(((((((..(((.(.((((.((((((...((((((((.........).)))))))...)))))).)))).).))))))))))).....).).)))))    reads   mm
    ........................cugugugguacucggagagau................................................................................    1       0
    .........................................agauaguagaccguauagcgu...............................................................    2       0
    .........................................agauaguagaccguauagcgua..............................................................    1       0
    ..........................................gauaguagaccguauagcgu...............................................................    1       0
    ..........................................gauaguagaccguauagcguac.............................................................    1       0
    ..........................................gauaguagaccguauagcguacg............................................................    4       0
    ...........................................auaguagaccguauagc.................................................................    3       0
    ...........................................auaguagaccguauagcg................................................................    3       0
    ...........................................auaguagaccguauagcgu...............................................................    189     0
    ...........................................auaguagaccguauagcgua..............................................................    295     0
    ...........................................auaguagaccguauagcguac.............................................................    4192    0
    ...........................................auaguagaccguauagcguacg............................................................    15278   0
    ...........................................auaguagaccguauagcguacgc...........................................................    67      0
    ...........................................auaguagaccguauagcguacgcu..........................................................    34      0
    ...........................................auaguagaccguauagcguacgcuu.........................................................    1       0
    ...........................................auaguagaccguauagcguacgcuuu........................................................    2       0
    ............................................uaguagaccguauagcgu...............................................................    26      0
    ............................................uaguagaccguauagcgua..............................................................    101     0
    ............................................uaguagaccguauagcguac.............................................................    2146    0
    ............................................uaguagaccguauagcguacg............................................................    7115    0
    ............................................uaguagaccguauagcguacgc...........................................................    69      0
    ............................................uaguagaccguauagcguacgcu..........................................................    74      0
    ............................................uaguagaccguauagcguacgcuu.........................................................    23      0
    ............................................uaguagaccguauagcguacgcuuu........................................................    6       0
    .............................................aguagaccguauagcguac.............................................................    1       0
    .............................................aguagaccguauagcguacg............................................................    6       0
    .............................................aguagaccguauagcguacgcu..........................................................    3       0
    .............................................aguagaccguauagcguacgcuu.........................................................    2       0
    ..............................................guagaccguauagcguacg............................................................    2       0
    ...............................................uagaccguauagcguacg............................................................    1       0
    .......................................................auagcguacgcuuuaucuguga................................................    1       0
    ..............................................................................guauguaacacgguccacuaac..........................    2       0
    ...............................................................................uauguaacacgguccacu............................    10      0
    ...............................................................................uauguaacacgguccacua...........................    21      0
```

```
                                                                               oar-miR-411a-3p
                                          oar-miR-411a-5p
ccccagccucuguccucggcgucccugugugguacucggagagauaguagaccguauagcguacgcuuuaucugugacguauguaacacgguccacuaacccucaguaucaaauccguccucgag

...............................................................................uauguaacacgguccacuaa..........................    86    0
...............................................................................uauguaacacgguccacuaac.........................    2244  0
...............................................................................uauguaacacgguccacuaacc........................    119   0
................................................................................auguaacacgguccacuaac.........................    1     0
................................................................................auguaacacgguccacuaacc........................    1     0
```
